# A randomised controlled trial of personalised decision support delivered via the internet for bowel cancer screening with a faecal occult blood test: the effects of tailoring of messages according to social cognitive variables on participation

**DOI:** 10.1186/s12911-015-0147-5

**Published:** 2015-04-09

**Authors:** Carlene J Wilson, Ingrid HK Flight, Deborah Turnbull, Tess Gregory, Stephen R Cole, Graeme P Young, Ian T Zajac

**Affiliations:** Flinders Centre for Innovation in Cancer, Flinders University of South Australia, Bedford Park, South Australia Australia; Cancer Council South Australia, Eastwood, South Australia Australia; Commonwealth Industrial Research Organisation, Food and Nutrition Flagship, Adelaide, South Australia Australia; School of Psychology, University of Adelaide, Adelaide, South Australia Australia; Telethon Kids Institute, University of Western Australia, Perth, Australia; School of Population Health, University of Adelaide, Adelaide, South Australia Australia

**Keywords:** Randomised controlled trial, Decision support, Bowel cancer, Faecal occult blood test, Cancer screening, Tailored messages

## Abstract

**Background:**

In Australia, bowel cancer screening participation using faecal occult blood testing (FOBT) is low. Decision support tailored to psychological predictors of participation may increase screening. The study compared tailored computerised decision support to non-tailored computer or paper information. The primary outcome was FOBT return within 12 weeks. Additional analyses were conducted on movement in decision to screen and change on psychological variables.

**Methods:**

A parallel, randomised controlled, trial invited 25,511 people aged 50–74 years to complete an eligibility questionnaire. Eligible respondents (n = 3,408) were assigned to Tailored Personalised Decision Support (TPDS), Non-Tailored PDS (NTPDS), or Control (CG) (intention-to-treat, ITT sample). TPDS and NTPDS groups completed an on-line baseline survey (BS) and accessed generic information. The TPDS group additionally received a tailored intervention. CG participants completed a paper BS only. Those completing the BS (n = 2270) were mailed an FOBT and requested to complete an endpoint survey (ES) that re-measured BS variables (per-protocol, PP sample).

**Results:**

FOBT return: In the ITT sample, there was no significant difference between any group (*χ*^2^(2) = 2.57, *p* = .26; TPDS, 32.5%; NTPDS, 33%; and CG, 34.5%). In the PP sample, FOBT return in the internet groups was significantly higher than the paper group (*χ*^2^(2) = 17.01, *p* < .001; TPDS, 80%; NTPDS, 83%; and CG, 74%). FOBT completion by TPDS and NTPDS did not differ (*χ*^2^(1) = 2.23, *p* = .13). Age was positively associated with kit return.

Decision to screen: 2227/2270 of the PP sample provided complete BS data. Participants not wanting to screen at baseline (1083/2227) and allocated to TPDS and NTPDS were significantly more likely to decide to screen and return an FOBT than those assigned to the CG. FOBT return by TPDS and NTPDS did not differ from one another (OR = 1.16, p = .42).

Change on psychosocial predictors: Analysis of change indicated that salience and coherence of screening and self-efficacy were improved and faecal aversion decreased by tailored messaging.

**Conclusions:**

Online information resources may have a role in encouraging internet-enabled people who are uncommitted to screening to change their attitudes, perceptions and behaviour.

**Trial registration:**

Australian New Zealand Clinical Trials Registry ACTRN12610000095066

## Background

In 2012 bowel (colorectal) cancer was the second most commonly diagnosed cancer in Australia for both men and women, and it was the third most common cause of cancer-related mortality in 2010 [1]. Participation in bowel cancer screening using a faecal occult blood test (FOBT) in accordance with recommended guidelines reduces bowel cancer mortality [2-4] and potentially incidence, although lowered incidence rates from use of FOBT alone has not been absolutely demonstrated [5,6]. Data on screening participation in Phase 2 of the Australian government-funded National Bowel Cancer Screening Program (NBCSP), which operated between July 1 2008 and June 30 2011, and provided free screening with an FOBT to people turning 50, 55 and 65 years, indicate sub-optimal uptake rates of 34.0%, 38.8% and 46.9% respectively [7]. Low participation rates in bowel cancer screening are widely reported and consistently evident across programs with different screening modes (e.g., colonoscopy and flexible sigmoidoscopy) and in different geographic locations [8-11].

Decision aids have been developed to assist consumers to optimise health-related decision making, including screening. These include written and audio materials, as well as interactive web-based tools. A recent systematic review has concluded that decision aids improve participants’ knowledge of the options available and perceptions that decision-making is informed [12].

Some evidence suggests that tailoring health information used in decision aids to the needs of the individual may facilitate desired changes in intention and behaviour, including cancer screening. Rimer and Kreuter [13] defined tailored health communication as “any combination of information and behaviour change strategies intended to reach one specific person based on information unique to that person, related to the outcome of interest, and derived from an individual assessment” (p. S184). A meta-analytic review of 56 tailored print health behaviour change interventions [14] reported a sample-weighted mean effect size of *r* = .074 (95% CI .066, .082). Although this can be characterised as approaching a small effect size, the impact of tailoring was largely moderated by demographic and behavioural variables and methodological features of the study. Importantly, the following were included among the variables that predicted a larger effect size; a focus on preventive or screening behaviour, recruiting participants from the household rather than an organisation, focusing tailoring on demographics and 4 or more theoretical concepts that predict behaviour, and using a social cognitive theory or stages of change model, or both, to guide tailoring.

Computer-based tailoring provides increased opportunity to deliver personal, tailored messages in a flexible, novel, timely and economically feasible manner [15]. A recent meta-analysis by Krebs, Prochaska and Rossi [16] confirmed that tailoring achieved benefits when information was delivered by computer. The study included 88 interventions that examined the impact of computer tailoring on smoking, physical activity, healthy eating and regular attendance at mammography. All four behaviours were positively impacted by computer-based tailoring with mammography adherence enhanced by 6% over the control condition. Although these results appear promising they failed to identify the nature of the information to be tailored, or the theories that might guide identification of the appropriate variables.

Albada, Ausems, Bensing, and van Dulmen [17] undertook a systematic review that assessed the impact of tailoring information about cancer risk and screening, concluding that more evidence was required before a positive impact from tailoring could be assumed. Moreover, the two studies included that examined FOBT use found only limited evidence of a beneficial effect from tailoring [18]. Consistent with this result, a recent attempt at establishing the efficacy of tailoring for people considering bowel cancer screening has had only limited success. Vernon et al. [19] undertook a randomised, controlled trial of a tailored, interactive intervention designed to enhance participation in a patient-selected bowel cancer screening option. In this study, messages received by the tailored on-line intervention group were determined by matching “process of change” feedback to the individual’s current stage of readiness to screen and preferred screening approach. Processes of change (e.g., “consciousness raising” as a process to move an individual from precontemplation to contemplation; see Prochaska, Redding & Evers [20]) were, in turn, mapped to hypothesised determinants (e.g., knowledge of the various forms of screening for bowel cancer) and a behaviour change technique [21]. Although exposure to the intervention material improved knowledge and self-efficacy for screening, these improvements did not lead to improved participation rates.

Although disappointing, these results may reflect tailoring on suboptimal variables. Myers et al. [22] tested both targeted (at those who were overdue for screening) and tailored invitations (messages that addressed personal barriers to screening) to increase screening participation. Tailored messages were developed on the basis of previously obtained scores on Preventive Health Model (PHM) variables (i.e., salience and coherence of screening, perceived susceptibility to bowel cancer, cancer worries and concerns, self-efficacy for screening, response efficacy for screening and perceived social support for screening) and matched to the individual’s decision stage for a stool blood test or a flexible sigmoidoscopy. Increased screening participation of between 11 and 15% was achieved for targeted and tailored interventions over usual care controls.

These promising results, together with the demonstrated positive effect of computer-based tailoring [16], encouraged us to develop an internet-based, tailored, Personalised Decision Support (PDS) tool for bowel cancer screening. Tailoring was based on the utilisation of individual responses to the PHM variables, previously validated for use in Australia [23], and their relationship to stage of readiness to screen in a manner directly comparable to Myers et al. [22]. An exploratory study to investigate the effectiveness of the PDS, whilst lacking statistical power, indicated the potential to positively address attitudes toward screening [24].

The protocol [25] for the current trial was developed after completion of the exploratory study. The aim was to compare the impact of communication materials tailored in real time and delivered over the internet to non-tailored material received in the same manner and mailed, non-tailored material (the latter approach being usual care for population screening in Australia). This design enabled us to investigate the contribution of message delivery mode (computer versus paper) and personal relevance of information (tailoring versus non-tailoring) or both, on participation. The primary hypothesis was that the internet-tailored information group would demonstrate higher return rates of FOBT compared to both internet and mailed non-tailored information groups. The secondary hypothesis was that internet-tailored information group would be at a higher stage of readiness to participate after the intervention and demonstrate greater improvements on PHM variables targeted on the basis of stage of readiness at baseline, and tailored according to need for motivation or reinforcement, also determined by responses at baseline, than participants in the other two groups.

## Methods

The methods described below summarise those in the published protocol [25] with the exception of two aspects. Firstly, the final sample size approached to recruit sufficient eligible persons was higher than that envisaged (~25,000 compared to the forecast of 18,000) due primarily to non-return of surveys and ineligibility arising because of recent participation in screening or endoscopic examination. Secondly, the protocol indicated that a secondary outcome of decisional conflict and satisfaction scores would be reported in addition to change on psychological variables. In the event, given the length and breadth of the current article, it was decided that these conflict and satisfaction outcomes should constitute a separate, future report.

### Study design and setting

Ethical approval for the study was obtained from the Commonwealth Scientific and Industrial Organisation Human Research Ethics Committee (09/33). The study was a parallel, randomised, controlled trial, stratified by sex and population density at the Australian state level and conducted in Australia from 2010 to 2012. A randomly-selected sample of men and women aged between 50 and 74 years from every mainland state who resided in urban electoral divisions was obtained from the Australian Electoral Commission roll. Electors whose addresses indicated that they were likely to be living in assisted accommodation (for example, an aged care hostel facility) were excluded from possible selection. Only one member from each household was included in the final sample to avoid possible cross-contamination between the groups. The family member retained was the one whose details came first in the supplied randomised list. The trial proceeded through a number of phases (Table 1). These are described in detail in the published study protocol [25] and summarised below.

**Table 1:** **Study interventions by phase and arm** ^**a**^

**Eligibility and Randomisation**	**Intervention**			**Evaluation**	
**Phase 1**	**Phase 2**			**Phase 3**	
Introductory letter Information sheet Eligibility Questionnaire (EQ) Pre-paid envelope	Group 1. Internet-based Tailored material (TPDS)	Information sheet; Baseline survey completed online (BS); Receipt of tailored messages; Electronic version of NBCSP consumer information booklet	FOBT kit mailed to those who completed BS; Reminder to revisit online tailored messages and NBCSP consumer information booklet	Endpoint Survey (ES) Completed Online	Telephone Interview (subset)^b^
	Group 2. Internet-based non-tailored material (NTPDS)	Information sheet; Baseline survey completed; online Electronic version of NBCSP consumer information booklet	FOBT kit mailed to those who completed BS; Reminder to revisit online NBCSP consumer information booklet	Endpoint Survey completed online	Telephone Interview (subset)^b^
Those returning EQ and meeting eligibility criteria randomised to 1 of 3 groups	Group 3. Paper-based non-tailored material (usual practice control group, CG)	Information sheet; Baseline survey completed on paper and returned to the researchers	FOBT kit mailed to those who completed BS; Printed version of NBCSP consumer information booklet	Endpoint survey completed on paper and returned	Telephone Interview (subset)^b^

^a^Adapted from Wilson et al. [25].

^b^These results published [33].

### Intervention phases

#### Phase 1: eligibility and randomisation

**Figure 1:**
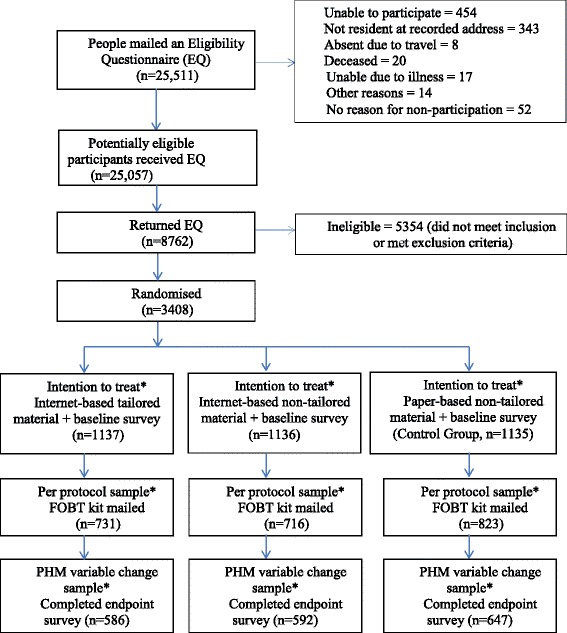
**CONSORT flow diagram** ^**a**^ **.** *The intention-to-treat analyses included all eligible participants who responded to the Eligibility Questionnaire and were randomised to a group regardless of further participation. In the Per Protocol analyses, we only included participants who completed a baseline survey and were mailed an FOBT. The PHM variable change sample constituted participants who completed an endpoint survey and contributed to analyses of change in baseline PHM variable scores following exposure to group allocation. ^a^ N = 63 participants, representing the 3 study arms and consisting of those who had both returned and not returned an FOBT, continued to a qualitative phase [33].

Following exclusion of those living in assisted accommodation or living in the same household as a previously selected elector, individuals remaining in the sample (*n* = 25,511) were mailed an introductory letter, an information sheet outlining the study, a short eligibility questionnaire (EQ) and a pre-paid return envelope. This sample was reduced to *n* = 25,057 after exclusions (Figure 1). The EQ addressed the inclusion criteria (aged 50 to 74 years inclusive; access to the internet) and exclusion criteria (FOBT screening within the previous 12 months; sigmoidoscopy or colonoscopy within the previous 5 years; clinical diagnosis of bowel cancer). Other data collected were demographic (employment status, education level), whether the internet was used to search for health-related information, willingness to receive unsolicited health information via the internet and source of internet access. The opportunity to win 1 of 3 $200 grocery-shopping vouchers was offered as an incentive to complete and return the questionnaire. Recipients were able to formally opt out of further correspondence at this stage. Return of the completed EQ was regarded as informed consent and willingness to participate in the trial, if eligible. Non-respondents were sent reminders at 4 and 6 weeks.

People meeting the eligibility criteria were stratified at the individual Australian state level and block-randomised using random allocation software [26] to one of 3 trial groups: internet-delivered Tailored Personalised Decision Support (TPDS) or Non-Tailored Personalised Decision Support (NTPDS), or a non-tailored, mail-delivered Control Group (CG). This sample provided the basis for the examination of treatment effects on screening participation using Intention-to-Treat analysis and is defined throughout as the ITT sample. Whereas investigators were aware of allocation status, participants and outcome assessors were kept blinded. Thereafter participants proceeded through a number of stages (Table 1) according to allocation, as described below.

#### Phase 2: Intervention

Two weeks following return of the eligibility questionnaire by the invitees, eligible participants were contacted. They were reminded that they could opt out at each point of contact with the project team. Those allocated to the TPDS and NTPDS groups were asked to access a website using a uniquely-allocated username and password. After logging in participants in both groups completed an on-line Baseline Survey (BS) that collected additional demographic data; Precaution Adoption Process Model (PAPM) decision stage [27]; Preventive Health Model (PHM) and self-efficacy variable scores, and a measure of faecal aversion (measures are summarised in Table 2). CG participants were mailed a paper BS, which they were asked to complete and return.

**Table 2:** **PHM, self-efficacy and faecal aversion variables; PAPM decision stages**

**PAPM decision stages for FOBT screening:**	
*Never heard of; Not considered; Decided against; Undecided; Decided to screen*	
**Factor**	**Statements**
**PHM** ^**a**^	
Salience and Coherence	Having colorectal cancer screening is an important thing for me to do^b^
	Colorectal cancer screening makes sense to me
	Having colorectal cancer screening can help to protect my health
	I will be just as healthy if I avoid having colorectal cancer screening^c^
Social Influence	Members of my immediate family think I should have colorectal cancer screening^b^
	I want to do what members of my immediate family think I should do about colorectal cancer screening
	My doctor or health professional thinks I should have colorectal cancer screening
	I want to do what my doctor or health professional thinks I should do about colorectal cancer screening
Cancer Worries	I am afraid of having an abnormal colorectal cancer screening test result
	I am worried that colorectal cancer screening will show that I have colorectal cancer
Perceived Susceptibility	Compared with other persons my age, I am at lower risk for colorectal cancer^c^
	The chance that I might develop colorectal cancer is high
	The chances that I will develop colorectal polyps are high^b^
Response Efficacy	When colorectal polyps are found and removed, colorectal cancer can be prevented^b^
	When colorectal cancer is found early, it can be cured
Self-efficacy	I think that doing the test would be easy for me^b^
	Finding time to do the test would be difficult for me^c^
	Completing the test correctly would be easy for me
Faecal Aversion	Collecting faeces for the purpose of bowel cancer screening is distasteful^b,c^
	Collecting faeces for the purpose of bowel cancer screening is unhygienic^c^
	Giving a sample of faeces to another person is embarrassing^c^

^a^Preventive Health Model (PHM) items reproduced from Tiro et al. [35].

^b^Statements used for tailored assessment.

^c^Items were reverse coded.

Participants who returned the BS within 12 weeks defined the sample utilised in the modelling of the link between psychological variables and screening participation. They are defined throughout as the Per Protocol sample (Figure 1).

The internet Tailored PDS (TPDS) group participants received messages about screening designed to either motivate a change in attitude, where this was not consistent with screening participation, or reinforce existing attitudes, where these were consistent. Messages pertaining to each PHM variable were presented in an order that accorded with the results of previous research. This prior research had demonstrated that addressing a specific subset of PHM variables at specific stages was associated with movement to a higher stage of readiness to screen as measured by the PAPM [28-30]. In our study the two PHM variables that Myers and colleagues [28] established were most strongly linked to movement between specific PAPM decision stages were given priority and presented as the first two motivating and/or reinforcing messages. The aim of message order placement was to exploit the primacy effect whereby information that is important for medical decision-making is presented first in order to ameliorate problems with short term memory [31]. The construction and delivery of the tailored messages has been described previously in greater detail [24]. In addition to receiving these tailored messages, participants had an opportunity to view an on-line version of the National Bowel Cancer Screening Program (NBCSP) consumer information booklet [32].

The internet Non-Tailored PDS (NTPDS) participants did not receive any tailored messages in response to the BS; they were offered access to the on-line NBCSP consumer information booklet only. Control Group (CG) participants were mailed an information sheet and a printed BS, which they were requested to complete and return in a supplied, pre-paid envelope. Consistent with usual care, the CG participants received an information booklet with the FOBT.

All participants were mailed an FOBT and associated materials two weeks following completion of the BS. The screening package consisted of: (a) two-sample FOBT (immunochemical FOBT, OC Sensor, Eiken Chemical Co. Ltd, Tokyo, Japan) with instructions, two sample collection sheets and a reply paid envelope; (b) a Participant Details form to confirm personal details and nominate a preferred doctor, with a simple section for participants to provide signed consent to obtain clinical follow-up reports if required. A research-scale bowel screening facility (Bowel Health Service) provided all FOBTs, test development and reporting services. CG participants received a printed version of the NBCSP consumer information booklet and those in the intervention groups were reminded that they could return to the website and revisit their tailored messages and the NBCSP information booklet (tailored PDS group); or the NBCSP information booklet only (non-tailored PDS group). The Bowel Health Service, unaware of group allocation, analysed the returned samples and informed the participant if the result was negative. Where a positive result was found, both the participant and their nominated doctor were informed and assisted if requested with clinical follow-up. A postal reminder was sent to those who did not return their completed FOBT within 6 weeks of despatch. Individual FOBT return data were relayed to the investigators as ID only.

#### Phase 3: evaluation

Participants were contacted by letter 1 to 2 weeks after receipt of their FOBT, or 12 weeks following the invitation to screen if an FOBT was not returned. They were asked to complete the Endpoint Survey (ES), either by returning to the online site (intervention groups) or completing the accompanying ES in paper format (control group). The ES re-measured PHM/PAPM variables and collected additional information relating to outcome measures. Return of the ES determined a participant’s eligibility for inclusion in analyses examining the impact of changes on psycho-social variables on participation in screening. This group has been termed the PHM variable change sample (Figure 1).

Phase 3 also encompassed a qualitative exploration of decision-making around bowel cancer screening. A series of focus groups and individual telephone interviews were conducted with *n* = 63 participants representing the 3 study arms, who had both returned and not returned an FOBT. The interviews revolved around the participants’ reasons for their decision to screen or not to screen. A description of the conduct and findings of this qualitative component has previously been published [33] and will not be addressed further in this paper.

### Materials

An overview of the materials developed for the study follows. A more detailed explanation of how the Baseline Survey (BS) component was utilised in conjunction with the message library has previously been published [24].

### Surveys

The baseline and endpoint surveys contained a series of statements reflecting Preventive Health Model (PHM) [34,35], self-efficacy and faecal aversion variables. Self-efficacy and faecal aversion were measured using statements derived from previous research regarding FOBT use [34,36]. These statements are provided in Table 2. All responses were measured on a 5-point Likert scale ranging from “strongly disagree” to “strongly agree”. The user’s stage of decision to screen as measured by the Precaution Adoption Process Model (PAPM, Table 2) was also ascertained in both surveys.

### NBCSP consumer information booklet

An electronic version of the NBCSP’s consumer information booklet [32] was developed. It provided the same information as that contained in the paper version save for information regarding how to opt off or suspend from the impending screening offer.

### Tailored internet intervention

Content received by the tailored group were the baseline and endpoint surveys for completion; a message library tailored to the individual user’s decision stage for screening and responses to PHM, self-efficacy and faecal aversion variables contained in the baseline survey, and access to an electronic version of the NBCSP consumer information booklet.

### Non-tailored internet and paper interventions

Materials for the non-tailored internet and paper groups were the baseline and endpoint surveys for completion and access to the NBCSP consumer information booklet (electronic or paper versions as appropriate).

### Statistical analysis

#### Sample size and power considerations

Sample size and power calculations were based on the numbers needed to test the primary hypothesised difference in participation between groups. A-priori calculations determined *n* =1080 participants per treatment group provided sufficient power (α = .05, β = .80) for a Chi-squared test to detect a difference of at least 10% between any two groups assuming a participation rate of 40% in the control group. Thus, the final number of *n* ≥1135 per treatment arm surpassed the requirements for adequate power.

### Statistical analyses

Data analysis was completed during 2014. Comparison of participation data between the three groups was performed at the Intention-to-Treat randomisation level (ITT Sample). Separate analyses were also undertaken on those participants who returned the baseline survey (Per Protocol Sample; *n* = 2270) in order to examine the impact of psychosocial factors on FOBT uptake. Univariate binomial Generalized Linear Models (GLMs) were conducted to assess the impact of the intervention on FOBT return in both the ITT Sample and Per Protocol Sample. The impact of the intervention was assessed by coding treatment arms according to two, two-level factors (internet vs. paper and tailored vs. non-tailored) in order to model main effects and an interaction. GLMs were then conducted that included all demographics and, for the Per Protocol Sample, scores for psychosocial constructs measured at baseline that were found to predict FOBT return in univariate models at the two-tailed *p* < .05 level. GLMs were also used to assess the impact of the intervention on FOBT uptake in participants distal to screening at baseline. As a secondary analysis, we examined change in the psychosocial variables over time. Change scores were calculated by subtracting baseline from endpoint scores in individuals who completed an endpoint survey (PHM variable change sample). Factorial ANOVAs were then used to assess the impact of trial allocation and FOBT return on change scores within this subgroup.

## Results

### Participant characteristics

Approximately 35% of people (8,762 of 25,057) returned the Eligibility Questionnaire (EQ). Of the respondents, significantly more: were from South Australia and Western Australia compared to New South Wales and Victoria (p = <.001); were from 60:64 and 65:69 age groups compared to the 50:54 group (p = <.001); and were female (p = <.001). Of the *n* = 8,762 who returned the completed EQ, *n* = 3,408 were identified as eligible (38%; 13.4% of those initially approached), randomised to intervention arms and defined the ITT sample: internet Tailored PDS (TPDS, *n* = 1137), internet Non-Tailored PDS (NTPDS, *n* = 1136) and paper-based non-tailored Control Group (CG, *n* = 1135). There was no significant difference between these groups in use of the internet to search for health information (p = .36) or willingness to receive unsolicited health information via the Internet (p = .62). A more detailed discussion of these latter results has previously been published [37]. All individuals in the ITT sample were mailed a Baseline Survey (BS) in order to progress to a screening offer. Of these individuals, 20 (<1%) BS packages were returned to sender, 250 (7%) opted out of further participation, 13 (<1%) returned a blank or significantly incomplete questionnaire, 855 (25%) did not return a survey or contact the researchers, and 2,270 (66%) returned a BS and formed the Per Protocol sample for further analyses.

Group characteristics were compared in each of the ITT and Per Protocol samples. As shown in Table 3, the groups were relatively homogenous with regards to demographics. Overall, the groups were mostly comprised of individuals aged 65 years and under and the proportion of males to females was relatively equal. According to the Socio-Economic Indexes for Areas (SEIFA) Index of Relative Socio-economic Advantage and Disadvantage Deciles [38], participants were mostly from a background with higher advantage indices.

**Table 3:** **Demographic characteristics of ITT and per protocol samples**

			**Control**		**Non-tailored**		**tailored**	
			**n**	**%**	**n**	**%**	**n**	**%**
ITT Sample	AGE	50–54	348	30.7%	330	29.0%	331	29.1%
		55–59	302	26.6%	298	26.2%	315	27.7%
		60–64	266	23.4%	296	26.1%	275	24.2%
		65–69	126	11.1%	123	10.8%	142	12.5%
		70–74	93	8.2%	89	7.8%	74	6.5%
	SEX	Male	557	49.1%	556	48.9%	559	49.2%
		Female	578	50.9%	580	51.1%	578	50.8%
	SEIFA*	SEI_AD <5	173	15.2%	173	15.2%	190	16.7%
		SEI_AD >5	962	84.8%	963	84.8%	947	83.3%
	STATE	NSW	219	19.3%	220	19.4%	220	19.3%
		QLD	214	18.9%	214	18.8%	215	18.9%
		SA	242	21.3%	242	21.3%	241	21.2%
		VIC	210	18.5%	209	18.4%	210	18.5%
		WA	250	22.0%	251	22.1%	251	22.1%
Per Protocol Sample	AGE	50–54	238	28.9%	207	28.9%	205	28.0%
		55–59	220	26.7%	187	26.1%	200	27.4%
		60–64	195	23.7%	177	24.7%	184	25.2%
		65–69	100	12.2%	85	11.9%	90	12.3%
		70–74	70	8.5%	60	8.4%	52	7.1%
	SEX	Male	413	50.2%	368	51.4%	371	50.8%
		Female	410	49.8%	348	48.6%	360	49.2%
	SEIFA*	SEI_AD <5	128	15.6%	97	13.5%	111	15.2%
		SEI_AD >5	695	84.4%	619	86.5%	620	84.8%
	STATE	NSW	154	18.7%	128	17.9%	123	16.8%
		QLD	162	19.7%	148	20.7%	139	19.0%
		SA	178	21.6%	151	21.1%	162	22.2%
		VIC	151	18.3%	129	18.0%	128	17.5%
		WA	178	21.6%	160	22.3%	179	24.5%

*SEIFA: <5 = higher disadvantage and lower advantage; >5 = lower disadvantage and higher advantage.

### Intervention effect on FOBT return

For the ITT sample, all individuals who did not return a FOBT, or who were not offered a FOBT for the reasons described previously, were regarded as non-participants. Overall screening participation rates were: internet tailored PDS (32.5%); internet non-tailored PDS (33%); and CG (34.5%). In the initial binomial GLM, treatment arm only was included as a predictor of screening participation and there was no significant effect of treatment arm on screening uptake as indicated by the overall test of model effects (*χ*^2^(2) = 2.57, *p* = .26). In a subsequent model we included demographics and, in this case, the overall model effects were significant (*χ*^2^(12) = 80.91, *p* = <.001; Table 4). Examination of model results indicates that age group had an effect on participation, with screening rates increasing with increasing age. Location differences were also evident with New South Wales residents less likely than Western Australia residents to return an FOBT.

**Table 4:** **Multivariate GLM of predictors of FOBT return utilising the ITT sample (N = 3,408)**

**Reference category**	**Comparison category**	**Odds-ratio**	**p-value**	**95% CI**	
				**Lower**	**Upper**
Control	Tailored	0.87	0.11	0.74	1.03
	Non-tailored	0.90	0.22	0.76	1.06
Female	Male	1.05	0.51	0.91	1.20
SEIFA - LOWER	SEIFA - HIGHER	1.16	0.14	0.95	1.41
Age 50–54 years	55-59	1.29	0.01	1.08	1.54
	60-64	1.67	0.00	1.39	2.01
	65-69	1.75	0.00	1.38	2.22
	70-74	1.97	0.00	1.49	2.62
WA	NSW	0.73	0.00	0.59	0.90
	QLD	1.13	0.25	0.92	1.40
	SA	1.19	0.11	0.96	1.48
	VIC	0.84	0.11	0.68	1.04

The same process as above was followed for the analysis of data from only those who returned a Baseline Survey, i.e. the Per Protocol Sample. Participation rates in this sample were as follows: internet tailored PDS (TPDS), 80%; internet non-tailored PDS (NTPDS), 83%; and paper-based control group (CG), 74%. In contrast to the ITT data analysis, an initial univariate model of the effect of the intervention on FOBT return showed a significant effect for the model overall (*χ*^2^(2) = 17.01, *p* < .001); screening participation in the internet groups (TPDS and NTPDS) was significantly higher than for the paper group (CG). A subsequent 2x2 chi-square comparing TPDS and NTPDS conditions showed these groups did not differ from one another (*χ*^2^(1) = 2.23, *p* = .13). A subsequent GLM model including all demographics was also significant overall (*χ*^2^(12) = 105.95, *p* < .001) and results are presented in Table 5. In this model, there were significant effects for treatment arm, sex, and age group. Consistent with the ITT analysis, increasing age was associated positively, although not linearly, to participation. Additionally, female gender was associated with FOBT return.

**Table 5:** **Multivariate GLM of predictors of FOBT return utilizing the per protocol sample (N = 2,270)**

**Reference category**	**Comparison category**	**Odds-ratio**	**p-value**	**95% CI**	
				**Lower**	**Upper**
Control	Tailored	1.36	0.01	1.07	1.74
	Non-tailored	1.69	0.00	1.31	2.18
Female	Male	0.81	0.04	0.65	0.99
SEIFA - LOWER	SEIFA - HIGHER	0.94	0.71	0.69	1.29
Age 50–54 years	55-59	1.53	0.00	1.19	1.98
	60-64	3.06	0.00	2.26	4.14
	65-69	2.22	0.00	1.55	3.18
	70-74	3.07	0.00	1.93	4.88
WA	NSW	0.81	0.20	0.60	1.11
	QLD	1.18	0.32	0.85	1.63
	SA	1.38	0.06	0.99	1.93
	VIC	0.86	0.35	0.63	1.18

### Psychosocial variables and FOBT return

In order to determine which psychosocial variables measured at baseline were associated with FOBT return, univariate GLMs were conducted with screening participation as the outcome variable. Descriptive statistics for psychosocial variables measured in the Baseline Survey (BS) are provided in Table 6. Salience & coherence, self-efficacy, faecal aversion and social influence were all significant univariate predictors (all p < .01), whereas cancer worries, perceived susceptibility and response efficacy did not predict FOBT return. A subsequent multivariate GLM was conducted that included treatment condition, the four significant psychosocial predictors, and demographic variables. This model was significant (*χ*^2^(12) =131.74, *p* < .001), but of the four psychosocial factors only self-efficacy remained statistically significant. Moreover, SEIFA did not contribute to the model and thus, the final model was revised again and is presented as Table 7. Higher levels of self-efficacy at baseline were strongly predictive of screening uptake and the effects of demographic variables remained consistent with the models presented previously. Treatment effects were also significant and consistent.

**Table 6:** **Descriptive statistics for baseline psychosocial variables in the Per Protocol Sample (N = 2,270)**

		**Control**	**Non-tailored**	**Tailored**	**Total**
Salience & Coherence	Mean	4.31	4.29	4.24	4.28
	SD	0.53	0.57	0.55	0.55
Cancer Worries	Mean	2.80	2.90	2.85	2.85
	*SD*	1.02	0.98	1.00	1.00
Perceived Susceptibility	Mean	2.80	2.89	2.82	2.83
	SD	0.54	0.57	0.53	0.55
Response Efficacy	Mean	3.72	3.78	3.74	3.75
	SD	0.59	0.53	0.55	0.56
Self-Efficacy	Mean	3.96	3.97	3.96	3.96
	SD	0.62	0.55	0.55	0.57
Faecal Aversion	Mean	2.43	2.45	2.46	2.45
	SD	0.86	0.84	0.81	0.84
Social Influence	Mean	3.48	3.68	3.63	3.59
	SD	0.75	0.68	0.66	0.70

Note: Correlations between variables were low, demonstrating relative independence: Mean *r* =0.11, Min *r* =0.01, Max *r* =0.30.

**Table 7:** **Multivariate GLM of predictors of FOBT return for per protocol sample (N = 2,270) including significant psychosocial variables**

**Reference category**	**Comparison category**	**Odds ratio**	**p-value**	**95% CI**	
				**Lower**	**Upper**
Control	Tailored	1.37	0.01	1.07	1.76
	Non-tailored	1.68	0.00	1.30	2.17
Female	Male	0.77	0.02	0.63	0.96
Age 50–54 years	55-59	1.49	0.00	1.15	1.93
	60-64	3.03	0.00	2.23	4.10
	65-69	2.09	0.00	1.45	3.01
	70-74	3.09	0.00	1.93	4.92
WA	NSW	0.81	0.20	0.59	1.12
	QLD	1.19	0.30	0.86	1.65
	SA	1.41	0.04	1.02	1.96
	VIC	0.86	0.34	0.62	1.18
Self-Efficacy	N/A	1.73	0.00	1.45	2.07

### Impact of intervention on participants uncertain about screening at baseline

It was hypothesized that the tailored intervention would improve decision stage more than the non-tailored approach. Ninety-eight percent of the Per Protocol Sample (2227/2270) provided complete data regarding their baseline decision stage and the distribution of individuals across these stages is provided in Table 8. About 15% of participants had never heard of screening for bowel cancer and about 28% had heard of screening but were not currently considering screening. However, the majority of participants in all conditions wanted to screen at study commencement (n = 1144/2227, or 51.4%). This is particularly relevant to the current trial, given that tailoring is concerned primarily with moving individuals who are not yet ready to screen towards actual screening behaviour rather than having an impact on those people who are already prepared to screen. To further explore the impact of tailoring on FOBT return, participants who already ‘want to screen’ were excluded (total n remaining for analysis = 1083/2227; TPDS = 351; NTPDS = 344; CG = 388) and a GLM was conducted in which trial condition in those not yet ready to screen was used to predict FOBT return. In addition to trial allocation, significant psychosocial predictors of screening in these individuals were identified through an iterative univariate and multivariate process and the final model is shown as Table 9. Results indicated that participants who were not ready to screen, and allocated to the internet PDS (tailored or not) were significantly more likely to complete an FOBT than those assigned to the paper-based control condition. FOBT return rates across all conditions in these individuals was as follows: TPDS 80% (279/351); NTPDS 82%; (282/344); CG, 70% (272/388).

**Table 8:** **Distribution of individuals across decision stages at baseline**

**Decision stage**	**n**		**Condition**		
	***%***	**Control**	**Non-tailored**	**Tailored**	**Overall**
Never Heard Of Screening		141	105	88	334
		*18.1%*	*14.7%*	*12.0%*	*15.0%*
Heard But Not Considered Screening		195	198	227	620
		*25.0%*	*27.7%*	*31.1%*	*27.8%*
Does not Want to Screen		7	3	2	12
		*.9%*	*.4%*	*.3%*	*.5%*
Unsure About Screening		45	38	34	117
		*5.8%*	*5.3%*	*4.7%*	*5.3%*
Wants to Screen		392	372	380	1144
		*50.3%*	*52.0%*	*52.0%*	*51.4%*
TOTAL		780	716	731	2227
					*100%*

**Table 9:** **Impact of intervention and psychological variables on FOBT return of participants (n = 1083) not yet ready to screen at baseline**

		**Odds ratio**		**95% CI**	
**Reference category**	**Comparison category**		***p*** **value**	**Lower**	**Lower**
Control	Non-Tailored	1.91	.001	1.276	2.621
	Tailored	1.64	.012	1.106	2.219
Salience & Coherence	N/A	1.38	.022	1.049	1.845
Perceived Susceptibility	N/A	.67	.006	.498	.891
Self-Efficacy	N/A	1.61	.000	1.264	2.128

Further examination of this model showed that the two internet arms did not differ significantly from one another with regards to screening participation (compared to tailored PDS, non-tailored PDS OR = 1.16, *p* = .42). In this sample, salience and coherence and perceived susceptibility, measured at baseline, predicted screening uptake. This result was not observed in previous analyses that included the Per Protocol Sample (see Table 7). The direction of the effect was as expected for salience & coherence, with higher scores associated with uptake, but it was opposite to what was found for perceived susceptibility; lower perceived susceptibility was associated with screening participation. Self-efficacy was again a significant predictor of screening participation in the expected direction, consistent with earlier analyses.

### Change on psychosocial variables

The hypothesis that scores on Preventive Health Model (PHM) variables would change following exposure to the intervention differentially according to group allocation was tested. All of those who completed the baseline survey (Per Protocol sample) were asked to complete the endpoint survey, and 80% did so (total *n* = 1,825/2270; internet tailored PDS 80% (586/731); internet non-tailored TPDS 83% (592/716); paper-based CG 79% (647/823). Baseline and endpoint scores for this sample are shown in Table 10. In order to explore change on these variables, change scores were calculated by subtracting baseline scores from endpoint scores. Using univariate ANOVAs, change scores were entered as the dependent variable and the effects of the independent variables Treatment Group (TPDS, NTPDS, CG) and FOBT return (Yes, No) and the interaction term (Treatment Group X FOBT return) examined.

**Table 10:** **Descriptive statistics for baseline and endpoint psychosocial variables for each condition and FOBT return group**

		**Salience & coherence**		**Cancer worries**		**Perceived susceptibility**		**Response efficacy**		**Self-efficacy**		**Faecal aversion**		**Social influence**	
		T1	T2	T1	T2	T1	T2	T1	T2	T1	T2	T1	T2	T1	T2
Control	**M**	**4.34**	**4.35**	**2.80**	**2.78**	**2.79**	**2.73**	**3.72**	**3.76**	**4.01**	**4.10**	**2.39**	**2.21**	**3.47**	**3.57**
	*SD*	*0.53*	*0.57*	*1.02*	*1.07*	*0.54*	*0.54*	*0.59*	*0.60*	*0.60*	*0.64*	*0.85*	*0.85*	*0.77*	*0.77*
	CI – Low	4.30	4.30	2.72	2.70	2.75	2.69	3.68	3.72	3.97	4.05	2.32	2.14	3.41	3.51
	CI – High	4.38	4.39	2.88	2.86	2.83	2.77	3.77	3.81	4.06	4.15	2.45	2.27	3.53	3.63
Non-Tailored	**M**	**4.30**	**4.35**	**2.88**	**2.83**	**2.87**	**2.82**	**3.79**	**3.78**	**3.99**	**4.11**	**2.45**	**2.17**	**3.70**	**3.67**
	*SD*	*0.56*	*0.56*	*0.99*	*1.08*	*0.57*	*0.60*	*0.52*	*0.68*	*0.54*	*0.66*	*0.84*	*0.85*	*0.68*	*0.66*
	CI – Low	4.26	4.30	2.80	2.74	2.83	2.78	3.75	3.73	3.94	4.06	2.38	2.10	3.65	3.62
	CI – High	4.35	4.39	2.96	2.91	2.92	2.87	3.83	3.84	4.03	4.16	2.52	2.24	3.76	3.72
Tailored	**M**	**4.25**	**4.38**	**2.84**	**2.77**	**2.81**	**2.88**	**3.74**	**3.80**	**3.98**	**4.15**	**2.43**	**2.06**	**3.65**	**3.74**
	*SD*	*0.54*	*0.55*	*1.01*	*1.12*	*0.53*	*0.61*	*0.55*	*0.68*	*0.56*	*0.63*	*0.82*	*0.82*	*0.65*	*0.65*
	CI – Low	4.21	4.34	2.76	2.68	2.77	2.83	3.70	3.75	3.93	4.10	2.37	2.00	3.60	3.68
	CI – High	4.30	4.43	2.92	2.86	2.85	2.93	3.79	3.86	4.02	4.21	2.50	2.13	3.71	3.79
FOBT Non-Return	**M**	**4.22**	**4.17**	**2.83**	**2.98**	**2.80**	**2.79**	**3.74**	**3.72**	**3.87**	**3.55**	**2.57**	**2.58**	**3.47**	**3.53**
	*SD*	*0.60*	*0.56*	*1.03*	*1.09*	*0.52*	*0.55*	*0.53*	*0.64*	*0.66*	*0.82*	*0.90*	*0.95*	*0.79*	*0.69*
	CI – Low	4.13	4.08	2.66	2.81	2.71	2.71	3.66	3.62	3.77	3.42	2.43	2.43	3.35	3.43
	CI – High	4.32	4.26	2.99	3.15	2.88	2.88	3.82	3.82	3.97	3.68	2.71	2.73	3.59	3.64
FOBT Return	**M**	**4.31**	**4.38**	**2.84**	**2.77**	**2.83**	**2.81**	**3.75**	**3.79**	**4.01**	**4.18**	**2.41**	**2.11**	**3.62**	**3.67**
	*SD*	*0.54*	*0.56*	*1.01*	*1.09*	*0.55*	*0.59*	*0.56*	*0.66*	*0.56*	*0.60*	*0.83*	*0.82*	*0.70*	*0.70*
	CI – Low	4.28	4.35	2.79	2.72	2.80	2.78	3.72	3.76	3.98	4.15	2.37	2.07	3.58	3.63
	CI – High	4.33	4.40	2.89	2.82	2.85	2.84	3.78	3.82	4.03	4.20	2.45	2.15	3.65	3.70

Results indicated that there was no effect of Treatment Group or FOBT return on amount of change in perceived susceptibility, response efficacy or social influence (all *p* > .14). For cancer worries there was a main effect (*F*(1,1819) = 8.17, *p* < .01, *η*^2^ = .004) for FOBT return only. Non screening participants showed an increase (*M* = 0.16, 95% CI [0.01, 0.31]) whilst screeners showed a decrease in cancer worries (*M* = −0.07, 95% CI [−0.02, −0.11]).

There were Treatment Group effects for salience & coherence (*F*(2,1819) = 5.59, *p* = .004, *η*^2^ = .01) and faecal aversion (*F*(2,1819) = 7.55, *p* = .001, *η*^2^ = .01). Perceptions about the salience & coherence of screening improved most in the tailored intervention group, followed by the non-tailored intervention group, and finally the control group. Specifically, Bonferroni post-hoc comparisons revealed a significant difference (*p* < .001) between the average change scores of the internet tailored PDS (*M* = 0.12, 95% CI [0.08, 0.17]) versus CG conditions (*M* = 0.01, 95% CI [−0.03, 0.04]), and also (*p* = .03) between internet tailored and non-tailored PDS groups (*M* = 0.05, 95% CI [0.00, 0.09]). The difference between the CG and non-tailored PDS was not significant (*p* = .65).

People in the tailored PDS group also showed greater declines in faecal aversion than the other two groups with a significant difference (*p* < .001) observed between tailored PDS (*M* = −0.37, 95% CI [−0.31, 0.42]) and CG conditions (*M* = −0.18, 95% CI [−0.12, −0.23]), but not (*p* = .12) between tailored PDS and non-tailored PDS (*M* = −0.28, 95% CI [−0.22, −0.34]). The non-tailored PDS group did however, exhibit greater decrease in faecal aversion than the CG (*p* = .03).

In addition to treatment effects, there was also a main effect from FOBT return on the variables salience & coherence (*F*(1,1819) = 8.35, *p* = .004, *η*^2^ = .01) and faecal aversion (*F*(1,1819) = 28.2, *p* = .001, *η*^2^ = .02). Those who screened reported greater improvements in the perceptions of screening salience and coherence (*M* = 0.07, 95% CI [0.04, 0.09]); those who did not return an FOBT showed little improvement (*M* = −0.05, 95% CI [−0.13, 0.03]). FOBT returners also showed a greater decrease in aversion to faecal sampling (*M* = −0.30, 95% CI [−0.28, −0.33]) than non-returners (*M* = 0.01, 95% CI [−0.09, 0.12]).

There were main effects of Treatment Group (*F*(2,1819) = 7.09, *p* < .001, *η*^2^ = .01) and FOBT return (*F*(1,1819) = 98.8, *p* < .001, *η*^2^ = .05) on self-efficacy. Participants in the tailored PDS condition showed significantly (*p* = .03) better improvement in self-efficacy (*M* = 0.17, 95% CI [0.12, 0.22] than CG participants (*M* = 0.08, 95% CI [0.03, 0.13]), but they did not show greater change than non-tailored PDS (*M* = 0.12, 95% CI [0.07, 0.17], *p* = .38). CG and non-tailored PDS groups were not significantly different (*p* = .99). Screening participants (*M* = 0.17, 95% CI [0.14, 0.19]) showed improvement whereas non-screeners displayed a worsening in their self-efficacy (*M* = −0.31, 95% CI [−0.20, −0.43]).

The interaction between treatment condition and FOBT return was also significant in this model (*F*(2,1819) = 4.77, *p* = .009, *η*^2^ = .01). An inspection of the means plot indicated that there was a generally consistent improvement in self-efficacy in all conditions for FOBT returners (CG: *M* = 0.14, 95% CI [0.09, 0.19]; non-tailored PDS: *M* = 0.17, 95% CI [0.12, 0.22]; tailored PDS: *M* = 0.21, 95% CI [0.16, 0.26]). By contrast, there was a more marked decrease in self-efficacy in non-screening non-tailored PDS participants (NTPDS: *M* = −0.59, 95% CI [−0.39, −0.79]) compared to the other conditions (CG: *M* = −0.33, 95% CI [−0.18, −0.48]; TPDS: *M* = −0.13, 95% CI [0.03, −0.29]).

## Discussion

Initial ITT analyses involving all those randomised, regardless of allocated group or whether they completed a Baseline Survey (BS) and received an FOBT, indicated no benefit on screening uptake from exposure to information about the importance of bowel cancer screening. Rates of participation varied little between groups, ranging between 32.5 and 34.5%, a rate comparable to the 33.5% most recently reported by the NBCSP for those invited between 2012 and 2013 [39]. Observed demographic differences between participants and non-participants were also largely consistent with those reported by the national program, although, unlike the NBCSP, neither gender nor socio-economic disadvantage had a significant effect on participation. Participation rates increased with age and differences between states were similar to published participation patterns.

By contrast, intervention effects were observed within the Per Protocol Sample (defined as those who completed a BS and were mailed an FOBT) in which participants exposed to online information provision returned FOBTs at a higher rate than those utilising paper-based information. This effect was also apparent in the reduced Per Protocol sample of individuals who at baseline were not ready to screen; this analysis excluded individuals who indicated in the baseline survey that they ‘*wanted to screen for bowel cancer’*. The difference between the results from the Per Protocol analyses and those of the ITT analysis suggests that people who complete a study questionnaire (i.e. the baseline survey) are in some way different from the broader sample, notwithstanding their demographic comparability. This difference may reflect differential engagement with the topic of bowel cancer screening and this may moderate the impact of any intervention. More specifically, individuals may need to have some degree of interest in the topic in order for an intervention to have an impact on behaviour.

Despite the contradictory results evidenced in the ITT and Per Protocol analyses, outcomes from the latter suggest that people in the recommended age group for screening who were connected to the internet, and who were prepared to participate in a questionnaire about bowel cancer, benefited from the provision of advice about the relevance of screening delivered via the computer. Screening rates varied significantly in the Per Protocol Sample with screening participation significantly higher for those who interacted with the screening materials online (tailored PDS and non-tailored PDS) rather than through the usual mail-based delivery (CG). These results did not, however, support online message tailoring as a strategy for incremental improvement of participation. Analysis of the impact of demographic variables on FOBT return highlighted again the generally positive relationship to age that was consistent with the ITT result. The only social cognitive variable to predict return in multivariate analysis of these data was self–efficacy for screening. This finding is largely consistent with other studies of tailoring including results from Vernon et al. [19].

The Per Protocol data results were also confirmed in analyses of intervention effectiveness that utilised only those in the sample who did **not** indicate a desire to screen at baseline, as measured by the PAPM. In this further reduced sample of people uncommitted to the targeted behaviour at baseline, provision of information about screening via the internet led to higher rates of FOBT return. The psychosocial predictor of FOBT return identified in the full Per Protocol sample, self-efficacy, was not influential when committed screeners were removed from the sample. Instead, salience and coherence of screening and perceived susceptibility to bowel cancer were influential; uncommitted participants who were more aware of screening salience and coherence at study start were more likely to be moved to act, and those with lower levels of perceived susceptibility at baseline were also more responsive to the intervention.

Results that analysed changes following the intervention on social cognitive variables of the Preventive Health Model and faecal aversion suggest that tailoring may influence some cognitions about screening. Consistent with Myers et al’s proposition that messages that improve people’s responses to critical social cognitive variables will impact intention and behaviour by moving them to a higher stage of readiness [22], our results confirm improvement on self-efficacy was a correlate of movement to action and improved more in those receiving tailored messages than in the control group, although not more than for non-tailored. Salience and coherence of screening was also positively impacted by tailored messages. Similarly, tailored internet-based information decreased faecal aversion more than mailed non-tailored information although not significantly more than non-tailored, internet-based information provision. Taken together with the analysis of non-committed participants, this result highlights the importance of making information about bowel cancer screening salient and coherent to potential participants, especially those not currently committed to screening. It is also important to note that failure to comply with the targeted behaviour (i.e. FOBT return) may negatively impact self-efficacy to act measured subsequently. This possibility is highlighted in the main effect of FOBT return on changes in self-efficacy. Moreover, the interaction effect suggests that, for non-participants, an internet-based, non-tailored message may exacerbate threats to confidence. An explanation for this would require further exploration.

There are both strengths and limitations to the current study. For example, the number of invitations required to achieve an eligible sample was notably higher than the initial proposed number (~25,000 versus 18,000). This was essentially due to a lower uptake rate for the eligibility phase and also the higher than expected incidence of self-reported screening in those who did return the eligibility survey, and these outcomes have been reported elsewhere [37]. The number of invitations was increased to ensure that we still recruited sufficient individuals per trial arm as governed by the power analysis. Despite the fact that there were significant demographic group differences (residence, age group, sex) between those who did and did not respond to the invitation, the eligible and randomised ITT groups were relatively homogenous with regard to these variables. An additional threat to generalisability was the study’s focus on internet-enabled participants; this compromises generalisability to population screening programs that include individuals who are not active internet users. However, this approach was necessary to ensure that internet access rates did not confound study results. Whilst a growing segment of the population within the targeted age-group is using the internet [40,41], there are still many sub-groups within the community without ready physical access, desire or capability to access the internet. Additionally, the study design, with the incorporation of survey return as a precondition for FOBT access, provided an additional hurdle to participation that is not observed in real-world screening programs. The significance of this threat is lessened by the observation that participation rates in the ITT sample were comparable to that obtained in the NBCSP.

The strength of the study rests with its multiple levels of analysis—from ITT to Per Protocol to subgroup analysis. Two of the three levels of analysis suggested that participation in screening may be enhanced in internet-enabled people by the use of internet-based communication. The incorporation of the measurement of change in psychological constructs provided the opportunity to analyse how message tailoring and mode of delivery impact upon attitudes and cognitions with some suggestion that the benefits of tailoring may accrue at this level. The implications of this require further examination; changes in the way people think about screening may impact ongoing participation.

## Conclusions

Internet-based information resources may have a role in encouraging some sections of the community who are uncommitted to screening to move along the decision pathway towards participation. Routine collection of these data in health practices might facilitate effective communication. Further research should examine the potential impact of social cognitive changes on subsequent participation in rescreening. It is possible that changes on important psycho-social influences on an intermittent behaviour like participation in biennial bowel cancer screening enhance perceived importance of screening and reduce dropouts from screening programs.
